# Cancer incidence and mortality in Brunei Darussalam, 2011 to 2020

**DOI:** 10.1186/s12885-023-10962-8

**Published:** 2023-05-22

**Authors:** Elvynna Leong, Sok King Ong, Khairil Azhar Si-Ramlee, Lin Naing

**Affiliations:** 1 Faculty of Science, Universiti Brunei Darussalam, Jalan Tungku Link, Bandar Seri Begawan, Brunei Darussalam; 2 Institute of Applied Data Analytics, Universiti Brunei Darussalam, Jalan Tungku Link, Bandar Seri Begawan, Brunei Darussalam; 3 NCD Prevention Unit, Ministry of Health, Commonwealth Drive, Bandar Seri Begawan, Brunei Darussalam; 4 PAPRSB Institute of Health Sciences, Universiti Brunei Darussalam, Jalan Tungku Link, Bandar Seri Begawan, Brunei Darussalam

**Keywords:** APC, Brunei Darussalam, Cancer, Incidence, Joinpoint, Mortality, Rates, Trends

## Abstract

This study presents the trends of age-standardised incidence and mortality rates of common cancers in Brunei Darussalam from 2011 to 2020. All cancer cases diagnosed among Brunei Darussalam citizens and permanent residents in the period 2011 to 2020 were included in the study. De-identified data were provided by the CanReg5 based BDCR, Ministry of Health Brunei Darussalam. The annual age-standardised incidence and mortality rates per 100,000 persons were standardised by the direct method using the World Health Organization (WHO) world standard population distribution. Joinpoint regression analyses were used to study the incidence and mortality trends of cancer in Brunei Darussalam over the 2011–2020 period. Trends were expressed as average annual percent change (AAPC) over 2011 to 2020, or annual percent change (APC) for a given time period. There were a total of 6,495 new cancer cases diagnosed and 3,359 death cases recorded from 2011 to 2020, in Brunei Darussalam. The five common cancers for males were colorectal, lung and bronchus, prostate, liver, and non-Hodgkin lymphoma. Among females, the five most common cancers were breast, colorectal, lung and bronchus, corpus uteri and cervix uteri. The five leading cancer deaths for males were lung and bronchus, colorectal, liver, prostate, and stomach, while for females, the five leading cancer deaths were breast, lung and bronchus, colorectal, ovary, and cervix uteri. There was a significant increase in the incidence trend of corpus uteri (AAPC$:13.3$) and a significant decline in the incidence trend for cervical cancer (AAPC$:-4.5$) from 2011 to 2020. There was a significant increase in the mortality trend of female breast cancer from 2011 to 2015 (APC$:16.3$), but the trend significantly declined from 2015 to 2020 (APC$:-12.5$). We also found a significant decrease in mortality trends for stomach cancer (AAPC$:-4.7$) from 2011 to 2020 for both genders combined. The burden of common cancers is expected to continue to grow with ageing population, effective public health interventions targeting high burden cancers and high-risk groups, and control of modifiable risk factors will continue to be the essential approaches in reducing cancer burden.

## Introduction

Globally, cancer is a major contributor to disease burden. There were an estimated 23.6 million incident cancer cases and 10.0 million cancer deaths across 204 countries and territories in 2019 [1]. The most commonly diagnosed cancers are breast, lung, colorectal, prostate, and stomach cancers globally [2]. Lung cancer is the leading cause of death, followed by colorectal, liver, stomach, and female breast cancers. Globally from 2010 to 2019, the number of new cancer cases and total cancer deaths increased by 26.3% and 20.9%, respectively [1].

In Brunei Darussalam, cancer is the principal cause of death. It accounts for about 19% of the total mortalities in the country [3]. The lifetime risk of being diagnosed with cancer in Brunei Darussalam is about one in four for men and one in three for women [4]. Brunei Darussalam’s population was estimated to be 453,600 (53.1% males and 46.9% females) in 2020, and the major ethnic groups are Malay (65.8%), Chinese (10.2%), and other ethnicities (24.0%) [5]. In 2019, it was reported that the lead cancer deaths in Brunei Darussalam among males were trachea, bronchus and lung cancer; rectum and anus cancers; liver and intrahepatic bile ducts cancers; prostate cancers; and non-Hodgkin lymphoma. Among females, the top five causes of cancer deaths were trachea, bronchus and lung cancer; breast cancers; rectum and anus cancers; liver and intrahepatic bile ducts cancers; and cervix uteri cancer [6].

In 2001, the Brunei Darussalam Cancer Registry (BDCR) was established by the Ministry of Health, to provide a population-based cancer database of all malignant cancers diagnosed in Brunei Darussalam. Cancer data were actively collected from the central pathology, hospital department medical records, and death registry as cancer reporting to BDCR is not mandated by law or regulations as notifiable disease in Brunei Darussalam. Medical practitioners or nursing professionals working in all the hospitals, health centres, and clinics, are encouraged to notify new cases of cancers to the BDCR using standardized notification form. In 2013, Brunei Darussalam implemented the national electronic patient record system called Brunei Healthcare Information Management System (BruHIMS). This has allowed for streamlining of data extraction and verification of malignant cancer cases, and enhanced the accuracy, timeliness, and completeness of the cancer registry [7].

Information on the change in cancer incidence and mortality trends is essential in the planning and evaluation of national cancer prevention and control programme. This study aims to estimate the incidence and mortality of common cancers in Brunei Darussalam from 2011 to 2020. Additionally, trends in cancer incidence and mortality for the five most common cancers during the same period were also compared.

## Materials and methods

All cancer cases diagnosed among Brunei Darussalam citizens and permanent residents in the period 2011 to 2020 were included in the study. De-identified data were provided by the CanReg5 based BDCR, Ministry of Health, Brunei Darussalam in a Microsoft Excel sheet. To strengthen data completeness, BDCR captured death data from the Registry of Births and Deaths obtained from the Department of Immigration and National Registration, any cancer-related deaths were reviewed and verified against medical records, Death Certificate Only (DCO) will be recorded if no other medical information is otherwise available. This study did not involve any direct contacts with patients.

Cancer incidence and mortality were stratified by gender, age group, and cancer site. All cancer cases were coded according to the International Classification of Diseases for Oncology, 3rd edition (ICD-0-3) and the tenth revision of International Classification of Diseases (ICD-10) [8]. Patients were categorized by gender (male and female) and ages were classified into 18 age groups: 0–4, 5–9, 10–14, 15–19, 20–24, 25–29, 30–34, 35–39, 40–44, 45–49, 50–54, 55–59, 60–64, 65–69, 70–74, 75–79, 80–84, and over 85 years. The cancer sites were lip, oral cavity, and pharynx (C00-10,12–14), nasopharynx (C11), oesophagus (C15), stomach (C16), colorectal (C18-20), liver (C22), gallbladder (C23), pancreas (C25), larynx (C32), lung and bronchus (C34), bone (C40-41), melanoma of skin (C43), breast (female) (C50), cervix uteri (C53), corpus uteri (C54), ovary (C56), prostate (C61), testis (C62), kidney (C64), bladder (C67), brain and central nervous system (CNS) (C70-72), thyroid (C73), Hodgkin lymphoma (C81), non-Hodgkin lymphoma (C82-85,96), leukemia (C91-95), and all cancers sites combined (C00-97).

### Statistical analysis

Age-specific incidence and mortality rates for all cancers were obtained by dividing the number of new cases and deaths in each age group by the corresponding population at risk, and multiplied by 100,000. The annual age-standardised incidence rates (ASIR) and mortality rates (ASMR) per 100,000 persons for the ten most common cancer sites and all cancer sites combined for each year were standardised by the direct method using the World Health Organization (WHO) world standard population distribution [9]. The statistical analyses were conducted using the R statistical software (Version 4.0.2).

Joinpoint regression analyses were used to study cancer incidence and mortality trends in Brunei Darussalam over the 2011–2020 period. The motivation for applying the joinpoint regression model lies in its ability to detect and quantify changes in the trends of incidence and mortality rates over time. We identified the time point(s) called joinpoints, when a change in the linear slope (on a log scale) of the temporal trend occurred, by connecting several different line segments. It is used to calculate trends by age-adjusted incidence and mortality rates for selected cancers. Trends were expressed as average annual percent change (AAPC) over the period 2011–2020, or annual percent change (APC) for a given time period, with 95% confidence intervals (CI). All APCs and AAPCs with their 95% CIs lying above or below zero, were regarded as increasing or decreasing trends, respectively. APCs and AAPCs with 95% CIs overlapping with zero were considered stable trends. The permutation test was performed to determine the minimum number of joinpoints necessary to fit the data. A significance level of 0.05 was used for the permutation test with 4500 of randomly permuted datasets [10]. This analysis was performed using the Joinpoint software (Version 4.9.1.0) from the Surveillance Research Program of the US National Cancer Institute [11]. We performed residual analysis and assessed the goodness of fit for our fitted joinpoint regression models, including normality and equal variance. The fitted joinpoint regression models in this study provided a satisfactory fit and adherence to the underlying assumptions. For all analyses, the level of statistical significance was set at 5%. Confidence intervals (CIs) of 95% were reported where appropriate.

Ethical approval for this study was obtained from the Medical and Health Research Ethics Committee of the Ministry of Health, Brunei Darussalam [Ref: MHREC/MOH/2022/1(1)].

## Results

### Cancer incidence

**Fig. 1:**
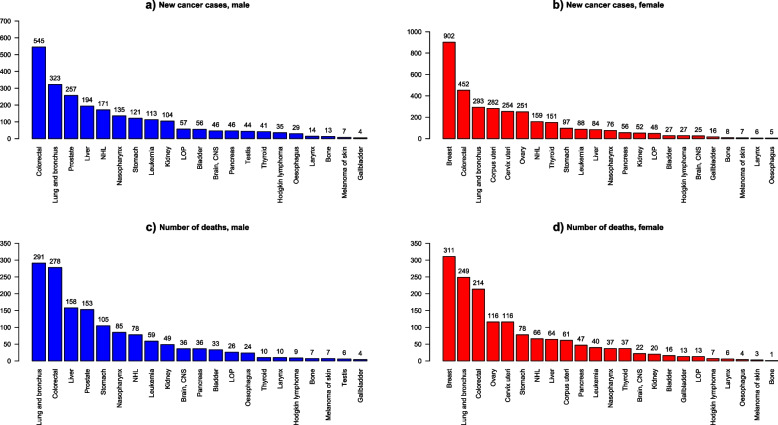
New cancer cases and deaths by gender from 2011–2020 in Brunei Darussalam NHL: Non-Hodgkin Lymphoma, LOP: Lip, oral cavity & phyarynx

A total of 6,495 new malignant cancer cases were recorded by the BDCR from 2011 to 2020 in Brunei Darussalam, where there were 3,792 female (58.4%) and 2,703 male (41.6%). The five most common cancers for male during 2011 to 2020 were colorectal ($$n=551;20.4\%$$), lung and bronchus ($$n=323;11.9\%),$$ prostate $$(n=258;9.5\%),$$ liver $$(n=195; 7.2\%)$$, and non-Hodgkin lymphoma ($$n=172; 6.4\%$$) (Fig. 1a). Among females, the five most common cancers were breast ($$n=906; 23.9)$$, colorectal ($$n=455; 12.0\%$$), lung and bronchus ($$n=293; 7.8\%$$), cervix uteri ($$n=286; 7.7\%$$) and corpus uteri ($$n=284; 7.5\%$$) (Fig. 1b).

**Fig. 2:**
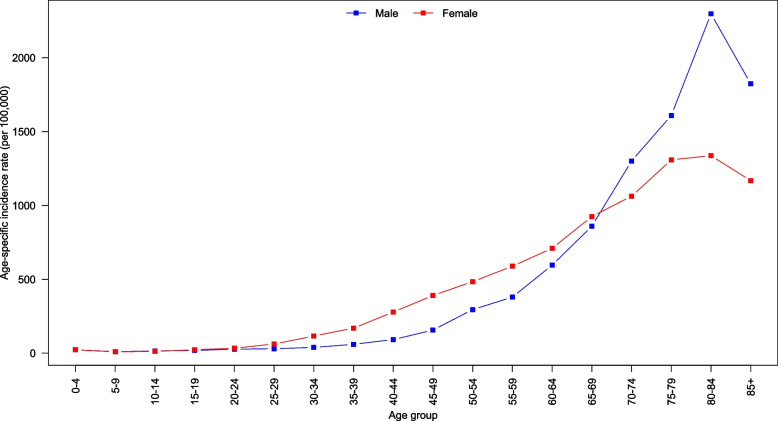
Age-specific incidence rates for all cancers by gender, 2011–2020

For females, the age-specific incidence rates for all cancers exhibited a gradual increase starting from the 25–29 years age group, reaching their peak at age 80–84 years age group. Similarly, for males, the age-specific incidence rates began to increase in the 35–39 years age group and also reached their peak in the 80–84 years age group. The rates declined after the age of 80–84 years old for both genders (Fig. 2).

**Fig. 3:**
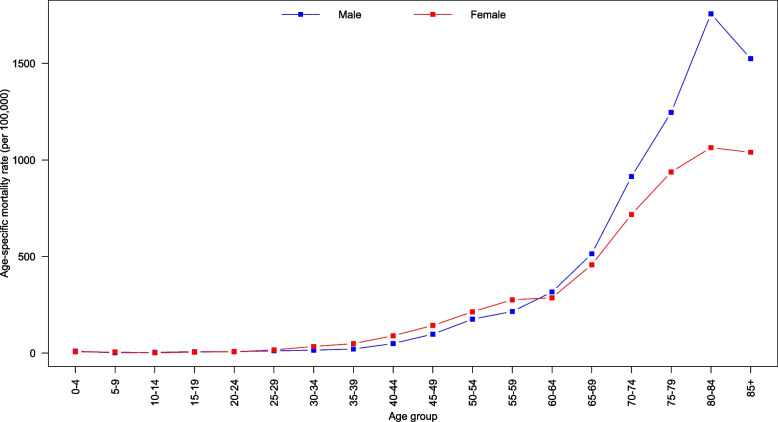
Age-specific mortality rates for all cancers by gender, 2011–2020

The age-specific mortality rates in Fig. 3 showed a steady increase for females, starting from the 30–34 years age group and continuing up to the 80–84 years age group. The rates declined for those aged 85 years and older. In contrast, for males, the rates started increasing in the 40–44 years age group, reaching a peak at 80–84 years before declining.

Tables 1 and 2 present the age-standardised incidence rates from 2011 to 2020 for the ten most common cancer sites and all cancer sites combined, for both genders combined, males, and females, respectively. In 2011, there were 516 incident cancer cases ($\text{A}\text{S}\text{I}\text{R}: 234.4$), but the cases increased to 827 newly diagnosed cases in 2020 ($\text{A}\text{S}\text{I}\text{R}: 230.5$). There were 211 males ($\text{A}\text{S}\text{I}\text{R}: 213.6$) and 305 females (ASIR$: 254.9$) newly diagnosed cases in 2011 but the number of cases increased to 342 males (ASIR$: 204.5$) and 485 females (ASIR:$254.9$) in 2020 (Tables 1 and 2).

**Table 1:** Age-standardised incidence and mortality rate for the ten most common cancers and all sites combined in males

Cancer site	Year									
	**2011**	**2012**	**2013**	**2014**	**2015**	**2016**	**2017**	**2018**	**2019**	**2020**
**Colorectal**										
ASIR	45.7	38.5	31.4	32.1	38.7	38.3	46.4	38.7	49.7	40.2
ASMR	26.4	27.3	21.7	16.4	12.0	23.7	30.1	26.7	26.3	12.6
**Lung and bronchus**										
ASIR	18.3	35.6	34.7	31.2	18.6	24.4	33.7	20.0	21.9	24.2
ASMR	28.7	23.5	29.3	28.2	16.7	25.1	29.2	16.9	26.8	22.2
**Prostate**										
ASIR	24.3	26.3	20.8	22.2	20.5	16.7	32.1	14.8	23.5	25.2
ASMR	18.1	18.3	20.5	15.9	7.9	7.4	21.3	15.2	17.2	17.7
**Liver**										
ASIR	13.2	17.2	14.2	16.4	17.6	8.0	14.4	14.2	13.1	14.3
ASMR	12.7	17.6	10.8	14.4	15.5	8.9	6.3	10.3	9.5	12.2
**Non-Hodgkin Lymphoma**										
ASIR	12.6	11.1	15.7	14.7	13.5	8.9	11.8	12.4	11.4	11.9
ASMR	5.6	6.1	9.6	8.6	7.9	4.8	6.0	5.2	7.8	3.6
**Nasopharynx**										
ASIR	13.5	11.8	10.7	3.7	8.8	9.6	7.8	10.7	5.6	9.9
ASMR	8.7	3.6	6.9	3.6	8.8	9.1	2.7	7.2	5.0	5.2
**Stomach**										
ASIR	14.7	9.4	8.2	7.9	7.3	8.1	11.0	10.6	10.1	10.3
ASMR	11.8	12.7	4.6	8.2	6.2	7.2	14.5	5.4	6.4	6.3
**Leukemia**										
ASIR	5.1	7.1	8.9	6.3	8.4	7.6	10.1	9.5	3.7	8.8
ASMR	2.6	1.8	3.7	3.7	2.2	4.0	7.6	4.0	6.6	5.2
**Kidney**										
ASIR	3.4	8.8	1.9	5.6	6.7	7.3	7.3	13.3	11.8	6.3
ASMR	0.7	2.0	1.8	2.4	4.1	5.7	6.5	2.8	7.1	2.0
**Lip, oral cavity & pharynx**										
ASIR	5.8	6.3	4.5	2.7	3.3	3.8	6.0	2.8	5.1	1.6
**Brain, CNS**										
ASMR	1.1	6.3	3.0	0.9	2.1	2.9	1.1	3.6	1.6	1.0
**All sites**										
ASIR	213.6	227.2	198.6	187.3	179.5	188.5	227.7	199.6	200.0	204.5
ASMR	142.8	150.6	142.8	129.9	111.9	124.1	168.9	118.6	151.4	103.6

*ASIR* Age-standardised incidence rate, *ASMR *Age-standardised mortality rate, CNS Central Nervous System

For males, colorectal cancer had the highest incidence rates in 2011 (ASIR:$45.7$), 2012 (ASIR$: 38.5$), and 2014–2020 varying from ASIR of 32.1 to 49.7. However, lung and bronchus cancer had the highest incidence rate in 2013 (ASIR$:34.7$). The second estimated highest incidence rates were observed in patients with prostate cancer in 2011 (ASIR$:24.3$), 2015 (ASIR:$20.5$), 2019 (ASIR: 23.5), and 2020 (ASIR: 25.2); colorectal cancer in 2013 (ASIR: 31.4); and lung and bronchus cancer in 2012 (ASIR$: 35.6$), 2014 (ASIR$: 31.2$), and 2016–2018 (ASIR$: 20.0-33.7$).

**Table 2:** Age-standardised incidence and mortality rate for the ten most common cancers and all sites combined in females

Cancer site	Year									
	**2011**	**2012**	**2013**	**2014**	**2015**	**2016**	**2017**	**2018**	**2019**	**2020**
**Female breast**										
ASIR	51.7	59.0	61.1	54.8	60.7	56.1	45.6	64.8	59.1	57.9
ASMR	17.5	16.3	19.9	22.2	28.6	25.7	23.0	19.9	15.9	14.0
**Colorectal**										
ASIR	29.8	31.8	40.8	27.5	30.7	28.0	22.5	29.8	34.1	33.0
ASMR	16.9	14.3	11.7	16.8	20.7	11.8	19.8	17.7	15.2	13.0
**Lung and bronchus**										
ASIR	15.8	33.5	29.3	28.2	33.4	20.0	11.2	22.2	20.0	17.8
ASMR	17.5	24.6	27.8	21.3	25.6	16.4	13.9	18.5	20.5	17.8
**Cervix Uteri**										
ASIR	19.3	19.9	20.4	16.8	18.9	15.4	16.6	18.2	13.0	12.2
ASMR	5.3	6.5	13.1	8.7	6.4	7.0	11.0	4.3	10.4	3.8
**Corpus uteri**										
ASIR	11.0	2.6	9.4	13.6	21.8	15.7	13.6	23.9	24.8	27.9
ASMR	0.0	2.4	0.0	2.6	4.5	7.0	4.8	4.7	7.5	3.8
**Ovary**										
ASIR	17.0	14.4	18.2	18.2	15.7	13.7	11.0	13.7	14.0	17.1
ASMR	6.2	7.7	10.6	10.2	10.4	8.4	6.6	4.4	8.9	5.0
**Non-Hodgkin Lymphoma**										
ASIR	13.1	7.5	16.7	6.9	9.6	10.6	9.6	13.1	11.4	9.8
ASMR	3.6	1.2	4.4	6.4	6.4	5.7	2.8	7.9	6.6	3.1
**Thyroid**										
ASIR	7.5	10.6	9.0	6.4	12.7	10.1	6.4	8.2	12.1	9.1
ASMR	3.3	1.9	1.8	2.6	2.4	2.6	3.3	1.1	3.5	4.3
**Stomach**										
ASIR	15.9	4.5	9.2	9.2	3.7	3.9	8.6	9.0	6.0	3.4
ASMR	11.2	3.4	8.3	8.8	5.5	4.9	3.4	8.0	5.7	3.3
**Liver**										
ASIR	1.8	3.7	12.1	7.2	4.5	4.6	4.5	6.2	12.0	3.6
ASMR	1.1	4.6	9.2	5.3	2.8	2.4	6.7	5.6	7.2	3.0
**Pancreas**										
ASMR	4.4	9.0	3.3	4.5	3.8	3.8	2.9	2.9	3.6	2.5
**All sites**										
ASIR	254.9	258.7	277.3	239.1	261.8	228.8	190.8	269.9	270.3	254.9
ASMR	121.4	118.4	136.7	138.8	141.7	122.4	121.6	116.9	137.3	94.7

*ASIR* Age-standardised incidence rate, *ASMR* Age-standardised mortality rate

For females, breast cancer had the highest incidence rates during 2011–2020 (ASIR$: 45.6-64.8$) followed by colorectal cancer in 2011 (ASIR$: 29.8$), 2013 (ASIR$: 40.8$), and 2016-2020 (ASIR$: 22.5-34.1$). Lung and bronchus cancer had the second highest incidence rates in 2012 (ASIR$: 33.5$), 2014 (ASIR$: 28.2$), and 2015 (ASIR$: 33.4$).

### Cancer mortality

There was a total of 3,359 death cases from 2011 to 2020, in which 1723 were female (51.3%) and 1636 were male (48.7%). The five leading cancer deaths for males in this study were lung and bronchus ($n=293; 17.7\%$), colorectal ($n=278; 16.7\%$), liver ($n=158; 9.5\%$), prostate ($n=153; 9.2\%$), and stomach ($n=105; 6.3\%$) (Fig. 1c). For females, the five leading cancer deaths were breast ($n=309; 17.8\%$), lung and bronchus ($n=250; 14.4\%$), colorectal ($n=214; 12.3\%$), ovary ($n=116$; 6.7%) and cervix uteri ($n=116; 6.7\%$) (Fig. 1d).

In 2011, 251 people died from cancer (ASMR$: 126.5$), and the number increased to 336 deaths in 2020 (ASMR$: 96.5$). Out of the 251 death cases in 2011, 127 cases were male (ASMR$: 142.8$) and 124 cases were female (ASMR$: 121.4$). In 2020, the death cases increased to 164 for male (ASMR$: 103.6$), while it was 172 for female (ASMR$: 94.7$) (Tables 1 and 2).

For males, the highest mortality rates were attributed to lung and bronchus cancer in 2011 (ASMR$: 28.7$), 2013–2016 (ASMR$: 16.7-29.3$), 2019 (ASMR: 26.8) and 2020 (ASMR$: 22.2$), while colorectal cancer had the highest mortality rates in 2012 (ASMR$: 27.3$), 2017 (ASMR: 30.1), and 2018 (ASMR$: 26.7$). Colorectal cancer had the second highest mortality rates in 2011 (ASMR$: 26.4$), 2013 (ASMR$: 21.7$), 2014 (ASMR$: 16.4$), 2016 (ASMR$: 23.7$), and 2019 (ASMR: 26.3). The second highest mortality rates were found in lung and bronchus cancer in 2012 (ASMR$: 23.5$), 2017 (ASMR$: 29.2$) and 2018 (ASMR: 16.9), while it was liver cancer in 2015 (ASMR$: 15.5$), and prostate cancer in 2020 (ASMR$: 17.7$).

However, for females, the highest mortality rates were observed in breast cancer patients in 2011 (ASMR: 17.5) and from 2014 to 2018 (ASMR: 19.9–28.6); lung and bronchus cancer patients in 2011 (ASMR: 17.5) as well, 2012 (ASMR$: 24.6$), 2013 (ASMR$: 27.8$), 2019 (ASMR: 20.5) and 2020 (ASMR$: 17.8$). The second highest mortality rates were observed in breast cancer patients in 2012 (ASMR: 16.3), 2013 (ASMR: 19.9), 2019 (ASMR: 15.9) and 2020 (ASMR: 14.0); lung and bronchus cancer patients in 2014–2016 (ASMR$: 16.4-25.6$), and 2018 (ASMR: 18.5); and colorectal cancer in 2011 (ASMR: 16.9) and 2017 (ASMR$: 19.8$).

### Trends

**Fig. 4:**
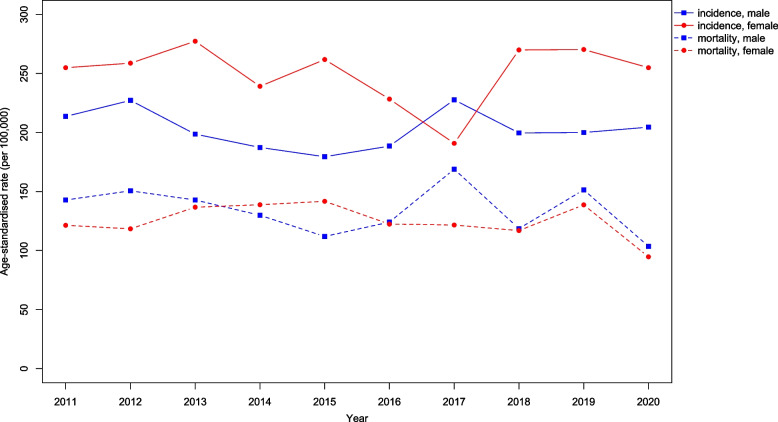
All cancer incidence and mortality (age-standardised rate) by gender in Brunei Darussalam from 2011 to 2020

**Fig. 5:**
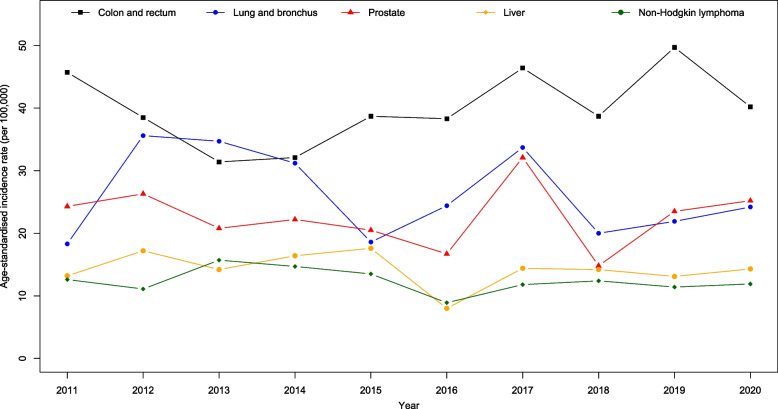
Trends of age-standardised incidence rates for five most common cancers in male

**Fig. 6:**
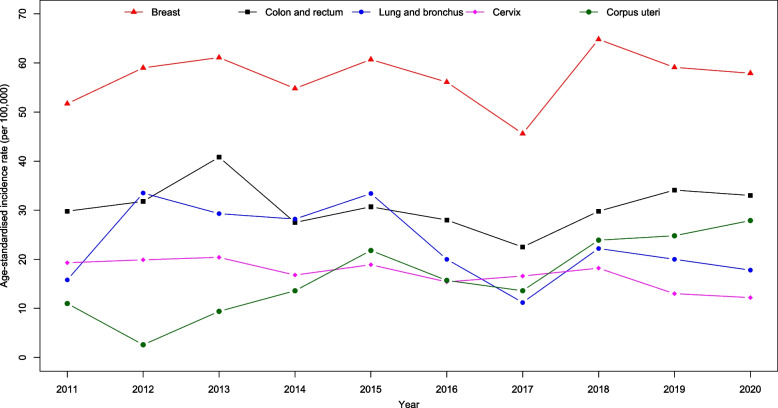
Trends of age-standardised incidence rates for five most common cancers in female

**Fig. 7:**
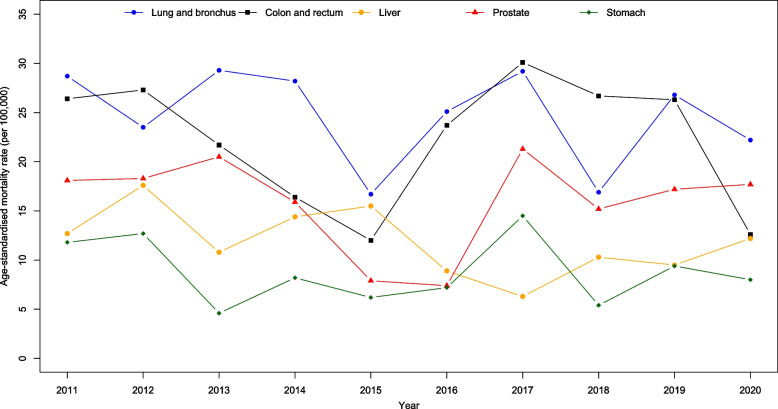
Trends of age-standardised mortality rates for five most common cancers in male

**Fig. 8:**
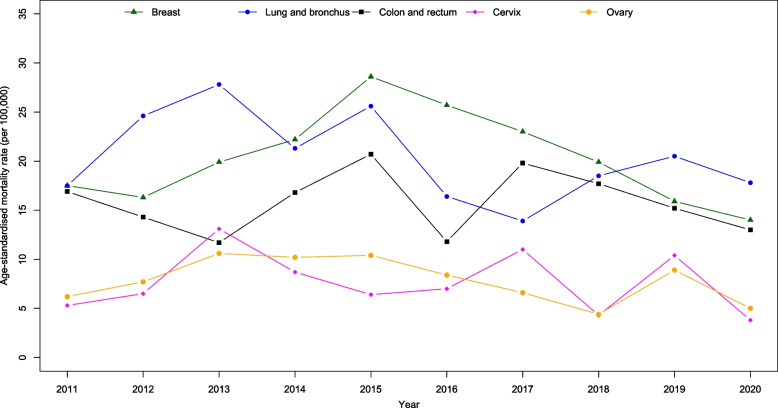
Trends of age-standardised mortality rates for five most common cancers in female

Figure 4 presents all cancer sites age-standardised incidence and mortality rates by gender from 2011 to 2020. We observed a big drop in incidence in 2017 for female and drop in mortality for both male and female in 2020. Joinpoint regression showed a significant decrease in the incidence trend for all cancers from 2011 to 2017 (APC$:-2.0; p=0.024$) and a significant increase from 2017 to 2020 (APC:4.5; $p=0.043$). However, mortality trends were reportedly stable ($p=0.171$). Figures 5, 6, 7 and 8 show the time trend for cancer incidence and mortality rates for the five most common cancers in males and females from 2011 to 2020.

### Incidence trend

Among males, all five most common cancers, which are colorectal, lung and bronchus, prostate, liver, and non-Hodgkin lymphoma, reported stable trends from 2011 to 2020 ($p>0.05$; Table 3; Fig. 5). Among females, there was a significant increase in the trend of the age-adjusted incidence of corpus uteri from 2011 to 2020 (AAPC$: 13.3; p=0.006$). However, we found a significant decline in the incidence trend for cervical cancer (AAPC$: -4.5; p=0.006$). Colorectal, lung and bronchus, and breast cancer showed stable trends in females from 2011 to 2020 ($p>0.05$; Table 3; Fig. 6).

**Table 3:** Age-standardised incidence and mortality trends for five most common cancers in males, females, and both genders combined

Cancer site	Male			Female			Both genders		
	Period	APC (95% CI)	*p*-value	Period	APC (95% CI)	*p*-value	Period	APC (95% CI)	*p*-value
**(A) Incidence rate**									
**All cancer site**	2011–2020	-0.2 (-2.3,1.9)	0.813	2011–2020	-0.2 (-3.0,2.7)	0.858	2011–2017 2017–2020	-2.1 (-3.6,-0.7) 4.6 (0.6,8.8)	0.014* 0.030*
**Colorectal**	2011–2020	1.8 (-1.8,5.6)	0.283	2011–2020	-0.3 (-4.4,3.9)	0.857	2011–2020	0.7 (-1.6,3.1)	0.482
**Lung and bronchus**	2011–2020	-3.0 (-9.2,3.7)	0.330	2011–2020	-5.8 (-13.3,2.4)	0.138	2011–2020	-4.4 (-9.1,0.4)	0.068
**Prostate**	2011–2020	0.3 (-5.3,6.2)	0.899						
**Non-Hodgkin lymphoma**	2011–2020	-2.1 (-5.9,2.0)	0.266				2011–2020	-1.6 (-5.6,2.5)	0.384
**Liver**	2011–2020	-1.5 (-6.6,3.9)	0.531				2011–2020	-0.9 (-7.0,5.5)	0.744
**Female breast**				2011–2020	0.5 (-2.1,3.2)	0.676			
**Cervix**				2011–2020	-4.5 (-7.1,-1.7)	0.006*			
**Corpus uteri**				2011–2020	13.3 (4.9,22.3)	0.006*			
**Stomach**							2011–2022	-4.2 (-10.7,2.8)	0.196
**(B) Mortality rate**									
**All cancer site**	2011–2020	-1.6 (-5.5,2.6)	0.406	2011–2020	-1.7 (-4.8,1.6)	0.267	2011–2020	-1.9 (-4.4,0.8)	0.147
**Lung and bronchus**	2011–2020	-2.0 (-7.0,3.2)	0.389	2011–2020	-3.4 (-8.5,1.9)	0.177	2011–2020	-2.8 (-5.6,-0.1)	0.055
**Colorectal**	2011–2020	-1.4 (-9.3,7.3)	0.718	2011–2020	-0.4 (-6.1,5.7)	0.892	2011–2020	-0.9 (-6.0,4.4)	0.690
**Liver**	2011–2020	-4.5 (-10.8,2.2)	0.157				2011–2020	-3.1 (-8.0,2.0)	0.197
**Prostate**	2011–2020	-0.7 (-9.2,8.6)	0.855						
**Stomach**	2011–2020	-2.0 (-10.4,7.3)	0.629				2011–2020	-4.7 (-9.2,-0.1)	0.047*
**Female breast**				2011–2015 2015–2020	16.3 (9.0,24.1) -12.5 (-15.8,-9.1)	0.002* < 0.001*			
**Cervix**				2011–2020	-1.8 (-12.5,10.2)	0.728			
**Ovary**				2011–2020	-4.5 (-11.6,3.2)	0.206			
**Non-Hodgkin lymphoma**							2011–2022	-1.1 (-8.9,7.3)	0.754

*APC Annual Percent Change, CI Confidence Interval*

*APC is significantly different from zero at $\alpha =0.05$

### Mortality trend

For males, although cancers such as lung and bronchus, colorectal, liver, prostate, and stomach showed declining mortality trends, joinpoint regression analysis showed stable trends from 2011 to 2020 ($$p>0.05;$$ Table 3; Fig. 7). For female, there was a significant increase in the mortality trend of breast cancer from 2011 to 2015 (APC$$:16.3;$$$$p=0.002$$), but the trend significantly declined from 2016 to 2020 (APC$$:-12.5$$; $$p<0.001$$). The mortality trends for females in lung and bronchus, colorectal, cervix, and ovary cancers were stable ($$p>0.05$$; Table 3; Fig. 8). However, for both genders combined, there was a significant decrease in the mortality trend for stomach cancer for both genders combined (AAPC$$: -4.7$$; $$p=0.047$$).

## Discussion

The observed age-specific incidence rates for all cancer highlight key differences between males and females, with both genders experiencing a steady increase in rates that peak at the 80–84 years age group. Age-specific mortality rates for females witnessed a consistent rise from the 30–34 years age group until the peak at 80–84 years, while males observed an increase beginning at the 40–44 years age group and peaking at 80–84 years. Following this peak, the rates declined for those aged 85 years and older. The observed differences between males and females may be attributed to various factors, including biological mechanisms, lifestyle factors, and screening practices. Moreover, the decline in both incidence and mortality rates after the age of 85 years may be related to several factors, such as competing risks from other age-related diseases, decreased cancer detection, and differences in cancer biology in older populations. Further investigation is needed to better understand the reasons behind this decline and to develop effective strategies for cancer management in older populations.

### Lung cancer

Lung cancer is the second most commonly diagnosed cancer and the leading cause of cancer deaths in this study. Over the 10-years study period, we found that lung cancer is the leading cause of cancer death in males, whereas in females, it ranks second for mortality after breast cancer. A recent study reported that globally, incidence and mortality rates are roughly two times higher in men than in women, although the ratio varies widely across regions [2]. The overwhelming majority of lung cancer is caused by tobacco smoking [12, 13]. Following the ratification of the WHO Framework Convention on Tobacco Control (FCTC), Brunei Darussalam has implemented several cost-effective tobacco control measures to curb tobacco use, such as increasing tobacco taxation, banning of advertisement or sponsorship, strengthening enforcement of smoke-free zones and expanding smoke-free zones, addressing illicit tobacco trade, and conducting mass media campaigns and education activities to prevent initiation of smoking and encourage smokers to quit [14]. Despite adoption of significant tobacco control measures, smoking prevalence has been found to be relatively high among men in Brunei Darussalam, with reports of tobacco brought in illegally. Almost one-fifth of the adult population in Brunei Darussalam were reported being current smokers, where males were close to ten times more likely to smoke than females [15]. A significant decrease in mortality trend was found in this study for both genders combined. The adoption of universal health coverage in Brunei Darussalam and availability of preventive services, early detection, referral and treatment of cancer are likely to have contributed to the reduction in lung cancer mortality observed in this study.

### Colorectal cancer

In this study, colorectal cancer ranks first in incidence and second in mortality. In comparison, colorectal cancer is the third most common cancer after lung and breast cancers globally [2]. The lifetime risks of developing colorectal cancer in Brunei Darussalam are higher than neighbouring countries such as Malaysia and Singapore [4]. Studies have shown that modifiable factors such as dietary patterns (low in fruits and vegetables and high in processed and red meat), sedentary lifestyle, use of tobacco, and intake of alcohol contributed to the increased risk of colorectal cancer [16–18]. It was reported that around 91.7% of Bruneian adults are not consuming the amount of fruit and vegetables recommended by the World Health Organization, and about a quarter of Bruneians are insufficiently active, where women are significantly more likely to be physically inactive compared with men [15]. In addition, about 72% of CRC cases were diagnosed in the regional or advanced stages [19]. To improve early detection and survival of CRC patients, Brunei Darussalam has implemented a national CRC screening program in 2019 allowing asymptomatic public aged 50 and above to be screened for CRC using faecal immunochemical test, in addition to opportunistic screening conducted by the clinicians [14, 19]. In addition, population-level programmes to encourage adoption of healthier lifestyle choices and participation of CRC screening. We did not find any significant changes in the incidence and mortality trends for colorectal cancer from 2011 to 2020. Future work will need to establish the impact of the screening programme on the cancer trends.

### Female breast cancer

We found that breast cancer had the highest incidence rates throughout 2011 to 2020, where 2020 recorded the highest incident cases. In 2019, the Ministry of Health implemented breast cancer screening as part of the national health screening programme [14], this could contribute to the increase in incident cases compared to earlier years. In our study, about 24.1% of cancer incidence among females were breast cancer. Studies have reported the association of breast cancer with lifestyle factors such as tobacco use, alcohol consumption, physical inactivity and unhealthy diet, and also excessive weight, high plasma glucose, hormonal and reproductive factors, as well as genetic predisposition. [20–23]. About 30% of Bruneian women were found to have obesity [15].

Breast cancer was found to be the leading cancer deaths for females in this study. There was a significant increase in mortality trend from 2011 to 2016, but the trend significantly declined from 2016 to 2020. This decline may be attributed to the combined effect of earlier detection and improved treatment [7]. Incidence and mortality rates for breast cancer vary considerably across geographic regions. Incidence rates were found to be higher in transitioned countries than in transitioning countries such as Australia/New Zealand, Western Europe, Northern America, and Northern Europe [2].

### Cervical cancer

Among Bruneian women, cervical cancer ranks fourth in incidence and mortality from 2011 to 2020. We found a significant decline in the incidence trend for cervical cancer (AAPC: -4.5). An organized national cervical cancer screening programme was initiated in 2011, and HPV vaccination has been provided to all female students of ages 10–17 years from government and private schools nationwide since 2012 [14, 24]. These interventions contributed to the significant decline in the mortality trend observed during the study period. The implementation of the national HPV vaccination programme, scaling up of the cervical cancer screening programme and strengthening of universal health coverage will continue the drive towards achieving the WHO’s 90-70-90 targets in the Global Strategy for the Elimination of Cervical Cancer a Public Health Problem by 2030 [25].

### Uterine cancer

Our study found that the incidence of uterine cancer continues to increase significantly during the study period, overtaking lung and cervical cancer as the third most common cancer among females in 2018, 2019 and 2020. Globally, cancer of the uterus is the sixth most commonly diagnosed cancer in women [2]. Increasing incidence of uterine cancer has been reported worldwide, particularly in higher-income countries and those undergoing rapid socio-economic transitions [26]. This increase might be attributable to high rates of obesity and physical inactivity, the two major risk factors in high-income countries [27] and other factors associated with uterine cancer include family history, use of hormones therapy, early age of menarche, diabetes, parity, metabolic syndrome, hypertension and polycystic ovarian syndrome [28].

### Prostate cancer

Prostate cancer was the third most common cancer and the fourth leading cause of cancer deaths among males in Brunei Darussalam. Our study did not find any significant changes in the incidence and mortality trends. Globally, prostate cancer is the second most frequent cancer and the fifth leading cause of mortality among men in 2020. Prostate cancer incidence rates in Asian countries are rising but remain significantly lower compared to Western countries [2]. Mortality rates trend were found to vary in Asian studies. South Korea observed an increase in mortality trend from 1983 to 2006 [29], while Singapore’s mortality rates declined 1998 to 2006 [30]. Countries such as Japan (year 2004–2013) [31] and China (2000–2009) [32] reported stable mortality rates, similar to our study.

The limitations of this study include underestimation of the cancer incidence and mortality data, in the earlier period of the cancer registry when data collection was done manually from paper medical records. The implementation of the national electronic medical records (BruHIMS) since 2013 has strengthened cancer surveillance. In addition, this study is limited to analysis of data on citizens and permanent residents of Brunei Darussalam. The exclusion of newly diagnosed cancer cases among temporary residents may not reflect the actual cancer burden and workload in the healthcare system. Routine physical examination of the human body plays a vital role in the early diagnosis of various cancers [33]. Future study could consider the use of machine learning models for precise cancer risk prediction to support screening and early detection of common cancers in Brunei Darussalam. Such models offer high-accuracy prediction techniques which allowed for individual stratification of prevention strategies and clinical management in personalized medicine [34, 35].

The study observed a significant drop in the ASIR for females in 2017 and ASMR for both sexes in 2020. There were a number of possible factors contributing to the decrease, these include introduction of new services including national screening programme, and also multidisciplinary cancer management services in the national cancer centre. In addition, our study period included the period during the global COVID-19 pandemic, further studies will be useful to estimate the impact of the pandemic on cancer incidence and mortality. Many preventive and routine medical activities, such as cancer screening and early detection programme, had to be postponed or ceased temporarily as hospitals and health centres grappled with infection control and manpower shortage, in addition to movement restrictions experienced by the general population [36]. The pandemic had also affected training of returning new medical officers in many clinical skills including ICD-10 coding and certification of death certification on the cause of death. Further evaluation study and continued cancer surveillance will be needed to ascertain the degree these factors impacted the disease burden and trends over time. In addition, future studies could consider building a prediction model using the available data, as this approach can provide valuable insights into cancer incidence and mortality trends, ultimately supporting public health efforts and improving patient outcomes.

This is the first study analysing changes over the past ten years for cancer incidence and mortality for common cancers in Brunei Darussalam. Prior epidemiological studies that were conducted for Brunei Darussalam mainly focused on specific cancer sites. Our study showed a significant increase or decline in the incidence and mortality trends of some cancers from 2011 to 2020. The burden of most cancers is expected to continue to grow as Brunei experiences an ageing population. Further research on cancer epidemiology, effective public health interventions targeting high burden cancers and high-risk groups, and control of modifiable risk factors will continue to be part of the essential approaches in reducing cancer burden.

## Data Availability

The data that supports the findings of this study are available from Ministry of Health Brunei Darussalam but restrictions apply to the availability of these data, and so are not publicly available. Data are however available from the corresponding author upon reasonable request and with permission of the Ministry of Health Brunei Darussalam.
